# Prevalence of sexual dysfunction among the male populations who seeking medical care for infertility, pregnancy loss and preconception care: a cross-sectional study

**DOI:** 10.1038/s41598-022-17201-3

**Published:** 2022-07-28

**Authors:** Xiaowei Yu, Songling Zhang, Zhentong Wei, XiaoYuan Zhang, Qun Wang

**Affiliations:** 1 Department of Reproductive Medicine, The First Hospital of Jilin University, Changchun, Jilin China; 2 Department of Prenatal Diagnosis, The First Hospital of Jilin University, Changchun, Jilin China; 3 Department of Obstetrics and Gynecology, The First Hospital of Jilin University, Changchun, Jilin China

**Keywords:** Epidemiology, Quality of life

## Abstract

The link between sexual dysfunction and male infertility has been well established. In addition to male infertility, male patients with couple pregnancy loss and preconception care are the most frequent reasons for the treatment of andrology outpatients. However, there is a paucity of information simultaneously investigating male sexual dysfunction in these males with different reproduction situations. A cross-sectional study was performed in consecutive series of 1256 participants, including 509 men with infertility, 437 couples with pregnancy loss, and 310 men for preconception care. All men completed a questionnaire on baseline demographic information, sexual behavior characteristics and validated research tools, including Premature Ejaculation Diagnostic Tool, seven-item Generalized Anxiety Disorder Scale, and International Index of Erectile Function. The prevalence of erectile dysfunction and premature ejaculation was 30.6%, 20.8% in the infertility population and 27.0%, 18.5% in pregnancy loss individuals, was much lower in preconception care men, at 9.3%, 11.9% (p < 0.05), respectively. Infertility and pregnancy loss couples were more biased toward choosing timed intercourse than preconception care couples, with rates of 19.6% in Infertility group and 17.4% in pregnancy loss groups, versus 10.0% (p < 0.05) in preconception care couples. The infertile and pregnancy loss men also reported higher rate of anxiety state than the preconception care group. The prevalence of erectile dysfunction increased gradually with the duration of infertility and the frequency of pregnancy loss, with a highest odds ratio of 7.346 (95% CI:4.329–12.467; P < 0.001) among men with ≥5 years of infertility, 6.282 (95% CI:3.446–11.453; P < 0.001) among couples ≥3 pregnancy loss when compared with preconception care group. The prevalence of erectile dysfunction, premature ejaculation and timed intercourse were comparable in pregnancy loss and infertile males, were all noticeably higher than preconception care group. There was also a trend toward a higher incidence of erectile dysfunction with longer duration of infertility or the more frequent of pregnancy loss.

## Introduction

Sexual dysfunction (SD) is common in the general male population. The most common male SD types are erectile dysfunction (ED) and premature ejaculation (PE), the combination of which affects approximately 30% of the adult male population and tends to increase in frequency with age^1^. Whether sexual function is normal or not is directly related to maintaining a good life quality in different domains, such as mental health, quality of relationships, and self-esteem, all of which has received considerable attention in studies over the years^2^. Much like how the Chinese proverb says “Appetite and lust are only natural”, procreation is not the sole purpose of human sexuality. However, sexuality is always linked to reproduction, sex pursued for the sole purpose of reproduction will be neither engaging or entertaining^3^. Thus, a correlation between unfulfilled fertility desires and SDs is observable in male partners of infertile and pregnancy loss(PL) couples^4–6^. Recent study also revealed that co-presence of both ED and PE was found in male partners of infertile couples^7^. PDE-5 inhibitors are effective in reversing stress-induced transitory impotence^8^, whereas men with fertility need are resistant to taking PDE-5 inhibitors for a variety of reasons.

Couples who visit clinics for help with reproduction issues often complain of sexual problems. Men who sought medical care are mainly divided into three groups; Infertility, defined as being unable to achieve a pregnancy within one year despite regular sexual intercourse and lack of contraception, affects 15% of couples and male-specific factors contribute to up to 25% of infertility^9^. PL is a spontaneous arrest of embryo development within 20–24 gestational week from conception, with two or more consecutive PLs known as recurrent pregnancy loss (RPL)^10^. Preconception care (PC), in this study refers to the couples who are in preparation or pursue a pregnancy for a duration not exceeding 1 year. The experiences of infertile men who have experienced recurrent failed conception attempts or men whose wives were clinically pregnant but later experienced pregnancy loss can result in a huge psychological burden. We were mindful of the potential morbidity of SDs on infertile and PL couples; Male partners of infertile couples are reported to suffer from psychological disorders and are at higher risk for SD^2,3^. Despite this, little information is currently available on the effects of PL on men^6^, and most previous studies have only focused on the female perspective^10^.

Infertility, PL and preconception care the most frequent cause of andrology department visits. To our knowledge, no study has examined the association between SD with male partners of infertile and PL in the same cohort. Therefore, this study aimed to investigate male SDs in male partners of infertile and PL couples simultaneously. In addition, the datas are compared to those male partners of preconception care couples rather than healthy control group with proven fertility, as they have a similar desire for fertility.

## Methods

The present study protocol was reviewed and approved by the Institutional Review Board of the First Hospital of Jilin University ethics committee (approval No. 21K064-001). All methods were performed in accordance with the relevant guidelines and regulations. Informed consent was obtained by all subjects when they were enrolled, with the ClinicalTrials.gov identifier (NCT number): NCT04941690. This investigation was a hospital-based, cross-sectional epidemiological study between June 2021 and October 2021, in which 1256 consecutive men were selected using the same criteria in the First Hospital of Jilin University. The collection of clinical datas followed the existing ethical guidelines and participants have all signed an informed letter of consent. Study sample size: the total number of participants for each study. Patients enrolled were: (i) greater than or equal to 20 years old and for the first time seeking medical care for couple infertility, either PL or PC. (ii) Men living together with wives and having regular intercourse during the study period; (iii) couples planning to try to conceive; (iv) men who voluntarily came to the andrology clinic to seek medical help. The exclusion criteria were: (i) men with a previous physician diagnosis of severe cardiovascular diseases, hypogonadism, and brain strokes; (ii) men separated with their wives or female factors prohibits sexual intercourse ≤1 time per month (such as during in vitro fertilization treatment, female surgical treatment or vaginal operation prohibits sexual intercourse); (iii) participants with diseases (such as diabetes mellitus, metabolic syndrome and severe hypertension) or medical conditions (such as antidepressants and antiandrogen) likely to cause SD were also excluded.

All participants completed a web-based questionnaire that included comprehensive demographic information in addition to the five-item version of the International Index of Erectile Function (IIEF-5) for diagnosis of ED, the Intravaginal Ejaculatory Latency Time (IELT) and Premature Ejaculation Diagnostic Tool (PEDT) for diagnosis of PE, and the 7-item Generalized Anxiety Disorder Scale (GAD-7) for anxiety, with a score of 5 or more suggesting anxiety. The IELT was measured from the initiation of vaginal penetration until ejaculation in the most recent attempt at sexual intercourse, was determined by the self-statement of the male participants. The Chinese versions of IIEF-5^11^, PEDT^12^, GAD-7^13^ have been validated. Since most of the male attended the clinic on his own, we were unable to evaluate the fertility and sexual function in females.

Severity of ED can be classified by the IIEF-5 scores as follows: a score of 22 to 25 indicates no ED, a score 17 to 21 indicates mild ED, a score 12 to 16 indicates mild- moderate ED, and a score 5 to 11 indicates moderate-severe ED^14^. Lifelong PE characterized by when ejaculation occurs it is always or nearly always within about one minute of vaginal penetration (LPE) or acquired PE is significant decrease in latency time usually does not last longer than 3 min (APE), an inability to delay ejaculation on all or nearly all vaginal penetrations and negative personal consequences^15^. A PE diagnosis is determined using a PEDT score of greater than 11, because this cut-score decreased the false-positive rate to 2.9% in a Chinese population^12^.

Timed (around the time of ovulation) intercourse (TI) is considered that more than 70% of sexual intercourse in a month is concentrated around the time of ovulation. Physical examinations mainly palpate for the presence and grade of varicocele or testicular parenchyma. To assess testes volume, a Prader orchidometer was used, all physical examinations were also performed by a solo physician. Samples of sperm were collected by masturbation in hospital after 3 to 5 days of sexual abstinence. Semen parameters, such as volume, concentration, total sperm count, motility and morphology, were measured according to the standard protocols of WHO criteria^16^. The predicted ovulation day using various ovulation prediction methods such as calendar charting, tracking basal body temperature, cervical secretion investigation, and urinary hormone measurement.

Datas were presented as the mean ± SD or percentage. Differences between more than two groups were assessed by using one-way ANOVA or Kruskal–Wallis test. Spearman’s, or Pearson’s, correlation test was used for assessing correlations and odds ratios (ORs) were determined using a logistic regression model and all adjusted for age. P-values < 0.05 were accepted as statistically significant. All statistical analysis was performed on SPSS (SPSS, Inc., Chicago, IL, USA) for Windows 22.0.

### Ethics approval and consent to participate

The study was approved by the First Hospital of Jilin University ethics committee (21K064-001) and participants signed informed consent.

## Results

Of 1336 men who sought medical care in our andrology clinic, 36 in the infertility group (6 patients refused participation; separated due to work:5; female factors prohibits sexual intercourse: 23; medications: 2), 39 in PL group( 20 patients refused participation; separated due to work: 3; female factors prohibits sexual intercourse: 16) and 5 in PC group (3 patients refused participation; separated due to work: 2) discontinued the study for reasons, resulting in a total of 1256 patients (mean age 32.5 years, range 21–52 years) who completed questionnaires and were included in the analysis between 26 June 2021 and 2 October 2021.

Table 1 lists the associations between the sociodemographics and main clinical characteristics. From these individuals, 509 were infertile men, 437 were male partners of couples with PL, and 310 were couples for PC. All sociodemographics and main clinical characteristics, such as mean testis volume and varicocele, were comparable. Only significantly decreased semen quality was present in infertile men.

**Table 1:** Sociodemographic and main clinical characteristics of included individuals.

Variable	Infertile (N = 509)	Pregnancy loss (N = 437)	Preconception care (N = 310)	P value
**Age, years**				
Mean ± SD	32.58 ± 4.40	32.64 ± 4.38	32.82 ± 4.23	0.744
Range	21–48	23–52	24–47	
BMI, kg/m^2^	25.93 ± 3.81	25.60 ± 3.44	25.94 ± 3.75	0.314
**Smoking**				0.190
Yes	245	190	154	
No	264	247	156	
**Alcohol**				0.509
Yes	100	95	71	
No	409	342	239	
**Night shifts**				0.217
No	405	364	253	
< 1 week/month	70	56	34	
1–2 week/month	30	12	19	
> 2 week/month	4	5	4	
**Marital status**				0.599
Primary	494	419	300	
Remarried	15	18	10	
**Education**				0.131
University	265	251	189	
High school	140	106	69	
Middle school	85	71	40	
Primary school	19	9	12	
Mean testis volume (ml)	13.19 ± 3.26	13.42 ± 2.75	13.27 ± 2.76	0.483
**Varicocele grade**				0.742
No	452	394	279	
Grade 1	18	20	14	
Grade 2	29	18	13	
Grade 3	10	5	4	
Semen volume, mL	3.62 ± 1.49	3.56 ± 1.35	3.56 ± 1.55	0.762
Sperm concentration, × 10^6^/mL	58.41 ± 47.73	68.69 ± 46.64	64.13 ± 48.88	**0.004**
Total sperm count, × 10^6^/mL	198.04 ± 165.17	232.95 ± 161.72	212.06 ± 162.83	**0.000**
Sperm progressive motility, %	30.74 ± 17.70	35.65 ± 18.77	33.40 ± 17.60	**0.000**
Sperm morphology, %	3.74 ± 2.05	4.01 ± 2.10	4.11 ± 1.89	**0.026**

Datas were expressed as mean ± standard deviation or number (percentage), when appropriate.

Datas were assessed with one-way ANONA or Chi-square test, when appropriate.

*BMI*  body mass index.

Significant values are in bold.

Table 2 depicts the characteristics of the participants, as well as the prevalence of SDs. The incidence of all kind of PE (PEDT score ≥ 11) were 20.8% in infertile men and 18.5% in PL men, which is significantly higher than the PC group rate of 11.9% (p < 0.05); Likewise, the highest prevalence of APE were observed in infertile group (13.3%) and PL group (12.8%), which were also significantly higher than the PC group rate of 7.4% (p < 0.05). The prevalence of ED (IIEF-5 score ≤ 21) was more common in infertile and PL men, existing in 30.5% of infertile and 27.0% of PL populations respectively. Meanwhile, the ED rate among PC men was only 9.3% (p < 0.05). And of this, compared to PC men, infertile and PL men also had higher subjective scores of anxiety.

**Table 2:** Sexual dysfunction, Sexual features, and anxiety among the subjects studied.

	Infertile (N = 509)	Pregnancy loss (N = 437)	Preconception care (N = 310)
**IIEF-5, scores**	21.99 ± 4.47*	22.67 ± 3.68*	24.15 ± 2.35
ED (%)	30.6*	27.0*	9.3
5–11	12.2	5.9	6.9
12–16	37.8	34.7	13.8
17–21	50.0	60.2	79.3
**PEDT, scores**	5.60 ± 5.12*	4.65 ± 4.99*	3.66 ± 4.66
PE (%)	20.8*	18.5*	11.9
APE (%)	13.3*	12.8*	7.4
LPE (%)	7.5	5.7	4.5
**IELT, min**	7.10 ± 5.54*	10.27 ± 7.69*	11.97 ± 8.08
Without PE	8.52 ± 5.38*	12.19 ± 7.25*	13.35 ± 7.62
ED	3.95 ± 3.90	6.34 ± 6.33	8.57 ± 8.16
Without ED	8.49 ± 5.59	11.75 ± 7.65	12.33 ± 8.01
**GAD-7, scores**	3.14 ± 2.88*	3.22 ± 2.54*	1.51 ± 1.79
Scores ≥ 5 (%)	24.4*	25.6*	6.5
**Timed intercourse (%)**			
Total	19.6*	17.4*	10.0
ED	41.0*	33.1*	17.2
Without ED	10.2	11.3	9.2
PE	40.6*	39.5*	13.5
Without PE	14.1	12.3	9.5

Datas were expressed as mean ± standard deviation or percentage, when appropriate.

*PL* pregnancy loss, *PC* preconception care, *PE* premature ejaculation, *APE* acquired premature ejaculation, *LPE* lifelong premature ejaculation, *ED* erectile dysfunction, *GAD-7* 7-item Generalized Anxiety Disorder Scale, *PEDT* premature ejaculatory diagnostic tool, *IIEF-5* International Index of Erectile Function, *TI* timed (around the time of ovulation) intercourse, *IELT *intravaginal ejaculatory latency time.

*p < 0.05 as compared with preconception care group.

Men disturbed by infertility and PL showed a much lower level of IELT, as the overall IELT in PC men was 11.97 ± 8.08, which was significantly higher in comparison to infertile men (7.10 ± 5.54) and PL men (10.27 ± 7.69) (p < 0.05). Suggesting that infertile and PL men displayed decreased aspiration for control at the time of ejaculation. Couples bothered with infertile and PL chose to allocate more sex to the time period of the “fertile window”, as TI was recorded for 19.6% of infertile and 17.4% of PL couples vs. 10% in PC couples (p < 0.05). Within this percentage, TI was found to take place in nearly 40% of infertile and PL men with SDs, whereas it was only present in less than 15% of men without SDs (p < 0.05).

According to the GAD-7 scale, 24.4% participants in infertile group and 25.6% participants in PL group had anxiety state, significantly higher than the PC group (6.5%, p < 0.01). The GAD-7 scores were also significantly higher in the infertile and PL group than in the PC group (Table 2).

Table 3 sought to evaluate associations between SDs and duration of infertility, frequency of PL, or anxiety. PEDT scores were positively associated with the duration of infertility (adjusted r = 0.168; P = 0.001), frequency of PL (adjusted r = 0.154; P = 0.012), and GAD-7 score (adjusted r = 0.472; P < 0.001). The IIEF-5score and IELT was negatively associated with the duration of infertility, frequency of PL, and GAD-7 score, all after adjusting for age.

**Table 3:** Associations between IIEF-5 scores, PEDT scores, IELT and duration of infertility, frequency of PL and anxiety.

	IIEF-5		PEDT		IELT	
	Unadjusted r, P	Adjusted r, P	Unadjusted r, P	Adjusted r, P	Unadjusted r, P	Adjusted r, P
Duration of infertility(year)	−0.218, P < 0.001	−0.178, P < 0.001	0.168, P < 0.0010	0.142, P = 0.001	−0.221, P < 0.001	−0.189, P < 0.001
Frequency of PL	−0.221, P < 0.001	−0.193, P < 0.001	0.145, P = 0.002	0.121, P = 0.012	−0.156, P = 0.001	−0.127, P < 0.001
Gad-7 scores	−0.543, P < 0.001	−0.528, P < 0.001	0.484, P < 0.001	0.472, P < 0.001	−0.377, P < 0.001	−0.360, P < 0.001

All adjusted by age.

*PEDT* premature ejaculatory diagnostic tool, *IIEF-5* International Index of Erectile Function, *IELT* intravaginal ejaculatory latency time.

**Figure 1:**
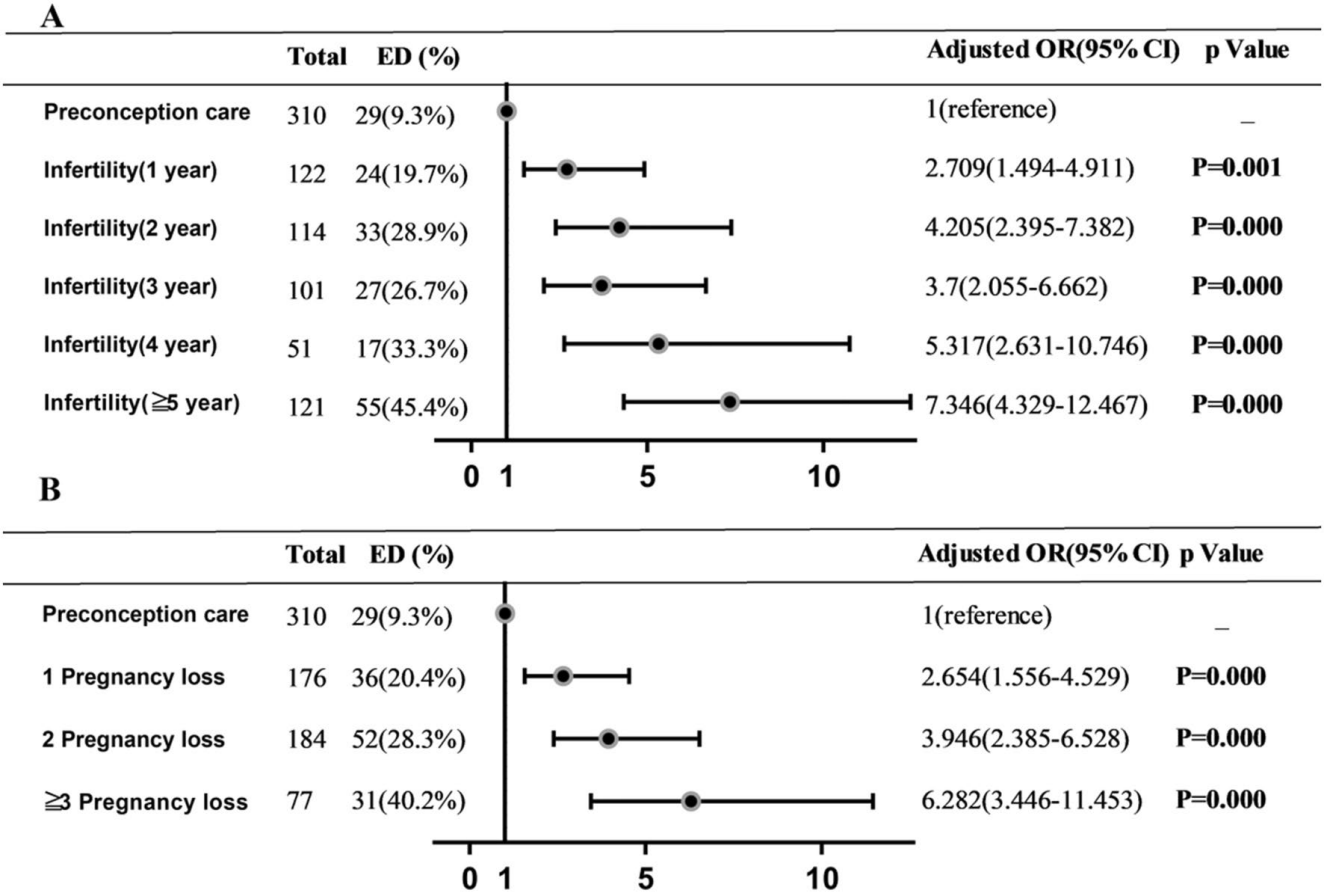
Multivariable logistic regression analysis of infertility duration (**A**) and PL frequency (**B**) associated with ED—all odds ratios in comparison to preconception care group and adjusted for age.

Figure 1 detail the ORs of ED occurrence through the duration of infertility and the frequency of PL, the PC group was used as baseline. The incidence of ED increases gradually with the duration of infertility and the frequency of PL, the highest ORs appeared in ≥ 5 years of infertility(adjusted OR = 7.346, 95% CI:4.329–12.467; P < 0.001) and frequency of PL ≥ 3(adjusted OR = 6.282, 95% CI:3.446–11.453; P < 0.001).

## Discussion

This is the first study investigating the male SDs in infertile and PL couples simultaneously, then comparing the results with those of subjects seeking PC of a similar age, with a similar need for fertility and without having suffered from infertility or PL. Pregnancy loss has been found to be fairly ubiquitous and was found in about 15% of women who have attempted to conceive. RPL occurs in approximately 2.5% clinically recognized pregnancies^10,17^, and, much like infertility, is a traumatic event, leading to symptoms of depression, anxiety, lowered self-esteem, and other psychosocial consequences^17,18^. According to our main results, in comparison with PC individuals, SDs were more prevalent in infertile and PL men and were closely associated with a increase in anxiety, duration of infertility, and the frequency of PL.

In this study, 30.6% of infertility men and 27.0% of men in PL group experienced ED, whereas it was only 9.3% in PC group. ED prevalence is similar to previous reports in infertile^4,19^ and PL^6^ couples in China. What is interesting is that we found couples who had experienced infertility and PL were more inclined to arrange their sexual lives during the ‘fertile window’. TI was seen in 19.6% of infertile men and 17.4% of men in PL couples, whereas in PC males this proportion is only for 10.0%. It was more pronounced in infertile men with ED (41.0%), PE (40.6%), or in PL individuals with ED(33.1%), PE(39.5%). ED is one of the most significant risk factors in a decrease in sexual intercourse frequency, as sex will rarely be spontaneous in men with SDs who more prefer TI (around the time of ovulation)^20^. Ultimately, this behaviour creates a vicious circle as those pursuing TI carry a higher risk of developing a SD^19,21,22^. Obligatory intercourse scheduled for a particular time separates sex from sexuality and can result in “situational ED”^22,23^. TI failure results in the most prevalent form of “situational ED”, many of these males only experience ED during their wives’ ovulatory time, dramatically increasing physical and psychosocial burdens^21,24^.

As tools for assessing PE varied may also no unified statement between the men and their partners^25^. The prevalence of PE (lifelong and acquired) varies by country: approximately in 25% of men in the United States, 20% in Germany and Italy, and 26% in Brazil^25^, and was also reported in11.2% of 3579 Chinese men^26^. In the current study, the prevalence of PE (lifelong and acquired) observed in PC men was 11.9%, which is similar to a general Chinese population(11.2%). Infertility (20.8%) and PL (18.5%) men also showed a higher but comparable frequency in our study. In this contribution, we show that the APE presents the highest incidence among participants in infertile group (13.3%) and PL group (12.8%), compared to 7.4% in the PC group. Lower IELTs were also identified among the infertile and PL population, where IELT was also negatively associated with the duration of infertility, frequency of PL and GAD-7 score. What intrigues us is that among those men without PE, the IELT in infertile and PL men was also significantly lower than that of PC men. This may be partly due to the fact that among men with reproductive problems, when sex is intended solely for procreation, sexuality was not simply due to sexual desire and the sex life was just to get the job done (intravaginal ejaculation)^3,27^.

There are studies that have evaluated associations between couple infertility/PL and psychological disorders^6,28^. In our cohort, the anxiety scores were much higher in men suffering from infertility and PL than for PC individuals. Men are usually immersed in the happiness of a pregnancy and do not anticipate a PL at all. Husbands sustained more anxiety when they perceive their partners suffered for PL, which may contribute to “situational ED” and fear of sex^29^.

This study had several limitations. First, we did not obtain complete information on the study participants, such as sex hormone profiles and the causes of male infertility, though some researchers believe that ED in young infertile men does not appear to be related to hormonal changes^30^. But it is widely accepted that androgens has a profound impact on general sexual function^31^. Second, it cannot be said for sure whether the individuals in this study accurately represent infertile, PL and PC men in general, as sexual function and anxiety may differ among men that visit an andrology clinic versus those not seeking medical help. In our sample, among men with an infertility period greater than five years, up to 15.7% (19/121) were never screened for fertility. This percentage was also as high as 33.8% (26/77) among couples with three or more PL. Predictably, many patients remain unscreened due to a go-with-the-flow optimistic attitude, not thinking that their infertility will be a problem. Therefore, as most of those who come to the clinic are couples who are already worried or anxious about their reproductive conditions, we may have overestimated the occurrence of SDs in these populations. Furthermore, as shown in previous study, the partner's Female Sexual Function Index scores were significantly associated with male partner sexual function. The coexist of sexual dysfunction in the female partner could also contribute to the deterioration of erectile function^32^. Unfortunately, in conservative cultures like China, most of the male attended the clinic on his own, also the sexuality, sexual health, and sexual functioning of women and their ability to express their attitudes and feelings toward it are still considered inappropriate. So we do not have the clinical datas available. Finally, the analyses were implemented cross-sectionally and a hospital-based, raising the possibility of selection bias.

## Conclusions

This is the first report to simultaneously assess the prevalence of SDs and related anxieties among infertile and PL men in China using PC males, who also have fertility need, rather than fertile men as controls. Based on the findings of this study, we should pay more attention to SDs in men of PL couples as well as infertile males, as the incidence of SDs were much higher in these men than PC couples. Men suffering infertility and PL were more like to choose TI, especially those men who are already experiencing ED or PE. They also present a shorter IELT than PC participants. Finally, the incidence of ED appears to increase with the duration of infertility and the frequency of PL.

## Data Availability

The datasets used and/or analyzed during the current study are available from the corresponding author on reasonable request.
